# The Week's Work

**Published:** 1911-01-07

**Authors:** 


### The Week's Work.

### HOSPITAL ADMINISTRATION AT THE CAPE.

We publish as a special article this week a summary of Sir Kendal Frank's interesting paper on the organisation and administration of hospitals in the Dominion of South Africa, as well as the main points in the discussion that followed the reading of the paper. Sir Kendal Franks is well known in this country as a distinguished surgeon: in Ireland he is also known and esteemed for the active personal interest that he took in hospital work. He is therefore, owing to his experience in Ireland and at the Cape, specially fitted to deal with the important question that he selected as the subject for his paper, and his remarks, dealing as they do with matters which are not of mere local interest, will be instructive reading to hospital men in this country as well as at the Cape. The animated discussion which the reading of the paper evoked shows that the medical profession in the Dominion is fully alive to the urgency of the whole matter.

### THE MEDICAL POINT OF VIEW.

One or two points call for special comment. Sir Kendal Franks laid stress on the fact that, from a professonal point of view, a hospital appointment at the Cape is not of such value as with us. In a country where specialisation has not yet advanced to the same extent as here, it is easy to see that the personal service which surgeons and physicians give to hospitals to which they are attached is greater, comparatively speaking, than it is with us in similar circumstances. The need for safeguarding the vital interests of the profession is therefore similarly greater. With us, as Sir William Collins has pointed out, there is an unfortunate tendency on the part of the public to regard protective—or let us say, defensive—action on the part of the medical profession as an attempt to obtain privileges and advantages not yet possessed. In a country like South Africa, where at present the profession is trying to promote solidarity and unity in its ranks, just as unity and solidarity have been effected in the political administration, no one can assert that medical men, in protesting against conditions which must in the nature of things affect them to their ultimate disadvantage, are "out for fees." For that reason the discussion on the several points raised in the paper is exempt from the objections that may legitimately be raised against similar discussions in which the lay interest in hospital administration and organisation has not been effectively represented.

### THE CAPE RESOLUTIONS.

The five resolutions passed by the Cape Congress will not perhaps receive the whole-hearted support of hospital men on this side. The first lays down the principle, with which no unprejudiced person is likely to disagree, that State aid is only for the really indigent. The second recommends the establishment of nursing homes for paying patients; while the third suggests the establishment of intermediate hospitals or special low-pay wards for the benefit of such patients as can afford only very small fees. The fourth resolution, however, admits the desirability of admitting patients, in centres where no private homes are in existence, into State-aided hospitals as paying patients, with the necessary safe-guarding clause so far as the staff is concerned. The last resolution deals with the question of certifying indigency, and recommends the institution of a special certificate from the medical practitioner as a voucher that the patient is really too poor to pay. We need not go into the question of paying wards in general hospitals—a matter which has at various times been fully ventilated in these columns—but we would merely point out that few nursing homes, however well equipped, can compete with a properly-managed modern hospital; and it seems to us that it is inadvisable therefore to limit the benefits such a hospital is able to give to indigent patients only, and to prevent patients who are able and willing to pay for the privilege of enjoying these benefits from entering the institution as paying patients. The arguments against paying wards in a general hospital brought forward in the general discussion are certainly not supported by the experience gained in institutions in this country, in America, or on the Continent. At Groningen, as we recently pointed out ("Hospital Relief in Holland," The Hospital, December 31, pages 419-420) the system of admitting three classes of private patients into the wards works admirably, and we venture to think that the conditions in Holland are, in some points at least, closely analogous to those obtaining in the Cape State. It appears to us that the purpose which the Congress had in mind in passing these resolutions would have been adequately served by the fourth resolution, so amended as to delete the words "where no nursing homes are available." Such a resolution establishes the definite principle, to which laymen as well as doctors would willingly subscribe, that the interests of the medical staff and the private practitioner should be efficiently safeguarded by requiring private patients to pay for medical treatment and advice.

### THE QUESTION OF INDIGENCY.

The fifth resolution raises rather an interesting side issue. Is a medical man justified in giving a certificate of indigency to a private patient, and—an even more important point—does he possess the right to advise a hospital almoner or board of the fact that a particular patient is able to pay for hospital treatment? At first glance it seems that the medical attendant has every right to do both, but it is well to bear in mind the fact that there is such a thing as professional secrecy, and that it is an axiom with medical men that they should not divulge information that comes to them in the course of their professional attendance on a case.

Where a patient specifically asks for such a voucher no objection can, of course, be entered; but the case is slightly different where a practitioner supplies a third party with information regarding the patient's ability or inability to pay. The question has, we believe, been raised here whether a general practitioner is justified in advising a hospital almoner with regard to a patient's means. The sense of the profession, as a whole, we fancy, is against permitting him to do so unless the patient consents to such information being given.

### HOSPITALS AND SCHOOL-CHILDREN.

There is a probability that considerable alterations will be made in the near future in the scheme favoured by the London County Council whereby grants are made to certain metropolitan hospitals for the treatment of children, certified as medically or surgically defective, who are attending the Council schools. The scheme, it will be remembered, has not been a conspicuous success. Complaints on both sides have been numerous, and not without solid foundation. The school doctors allege that the treatment of many of the patients sent up to hospitals is slipshod in the extreme and highly unsatisfactory. Dr. Kerr's last annual report gave some striking proofs of the truth of this allegation as regards one large metropolitan hospital at least. On the other hand, the hospitals allege, with equal truth, that the present system of inspection throws far too much work on the staff and that many of the patients sent up for treatment are not cases suitable for hospital care at all.

### SCHOOL-CHILDREN AND OUT-PATIENT DEPARTMENTS.

One large hospital has now, we understand, decided to refuse the grant and to revert to the old position. In that case there is little doubt that it will still have school-children to attend to. There is no obligation on a parent to take his child to a particular hospital. The children will therefore continue to attend as out-patients at various hospitals and will from time to time be seen by the school doctors. The results, we expect, will be the same. Complaints will continue to be made about the treatment, and under the present system of out-patient treatment, especially at hospitals where medical schools are primarily considered, such complaints are bound to have a justifiable basis. Out-patient treatment, as a whole, is in need of reform: it is certainly in need of attention. If a systematic examination were made of all cases discharged from or under treatment at an out-patient department at any large metropolitan hospital, the odds are that independent examiners would discover a good many cases of indifferent treatment. It is this systematic examination by outside examiners that has focussed public attention on the defective treatment given to school-children. There can be no question of wilful neglect on the part of hospital staffs so far as these patients are concerned: they are treated in the same manner as any other children who attend for advice and treatment. The difficulty lies in the out-patient department itself, and unless the Continental system of the Polyclinic is adopted we are bound to have unsatisfactory results so long as we continue along the old lines.

### THE FUTURE.

It is sincerely to be hoped that a frank discussion of the whole subject will be opened at an early date. The British Hospitals Association, in conjunction with the Hospital section of the British Medical Association, is in a position to initiate such discussion, and the benefits, both to institutions and to the community, from such a conference ought to be great. No teaching hospital can afford to regard with equanimity the establishment of special school clinics in its neighbourhood. No friend of the hospitals can afford to see the establishment of such clinics without an attempt to secure some scheme of co-operation whereby existing centres of treatment may be utilised. Yet, unless there is an alteration for the better in the arrangements of the out-patient department, the school clinic must come, with the concomitant result that the hospitals will lose prestige and, to some extent at least, public support. It is the attitude of the man in the street that counts for something in discussing this matter. He has no inside knowledge of the working of hospitals; he does not read institutional journals, but he does hear platform speeches in which, it is certain, much stress will be laid on the fact that the voluntary hospitals have been found inadequate—some will not scruple to say incompetent—to deal with school-children. Institutional workers who are aware of the difficulties of the position, and in a position to appreciate them, may know the error of such judgments, but they cannot merely smile at it. What is wanted is a thorough ventilation of the whole matter with the object of devising some practical way out of the *impasse*. At any rate, the difficulties which have been made public so far, and the comments which have been indulged in on both sides, should serve to rivet the eyes of institutional workers on the whole question of the out-patient department and the urgent necessity for its reform.

### THIS WEEK'S DRUG MARKET.

Considering the fact that most firms are still busy taking stock, business is quite as brisk as could be expected, and although the orders have, for the most part, been for small quantities, the tone of the drug market is healthy and the outlook promising. Cocaine hydrochloride is 7d. per ounce dearer. An advance in the price of American peppermint oil is not improbable. Very high prices are still quoted for olive oil, and there is no indication that prices are likely to recede for some time to come. A further advance in the price of santonin may take place at any moment. Strophanthus seed is tending dearer. The value of ipecacuanha is well maintained. Ergot of rye seems likely to continue to advance in price. The price of opium is fully maintained, and an advance is expected when the demand improves. No further announcement has been made by makers of bromides, but higher quotations are still expected.

